# WDR72 regulates vesicle trafficking in ameloblasts

**DOI:** 10.1038/s41598-022-06751-1

**Published:** 2022-02-18

**Authors:** Kaitlin Katsura, Yukiko Nakano, Yan Zhang, Rozana Shemirani, Wu Li, Pamela Den Besten

**Affiliations:** Department of Orofacial Sciences, School of Dentistry, University of California, San Francisco, 521 Parnasus Ave, Box 0422, San Francisco, CA 04143-0422 USA

**Keywords:** Cell biology, Diseases, Medical research

## Abstract

As the hardest tissue in the human body, tooth enamel formation is a highly regulated process involving several stages of differentiation and key regulatory genes. One such gene, tryptophan‐aspartate repeat domain 72 (*WDR72*), has been found to cause a tooth enamel defect when deleted or mutated, resulting in a condition called amelogenesis imperfecta. Unlike the canonical genes regulating tooth development, WDR72 remains intracellularly and is not secreted to the enamel matrix space to regulate mineralization, and is found in other major organs of the body, namely the kidney, brain, liver, and heart. To date, a link between intracellular vesicle transport and enamel mineralization has been suggested, however identification of the mechanistic regulators has yet to be elucidated, in part due to the limitations associated with studying highly differentiated ameloblast cells. Here we show compelling evidence that WDR72 regulates endocytosis of proteins, both in vivo and in a novel in vitro ameloblast cell line*.* We elucidate WDR72’s function to be independent of *intracellular* vesicle acidification while still leading to defective enamel matrix pH *extracellularly.* We identify a vesicle function associated with microtubule assembly and propose that WDR72 directs microtubule assembly necessary for membrane mobilization and subsequent vesicle transport. Understanding WDR72 function provides a mechanistic basis for determining physiologic and pathologic tissue mineralization.

## Introduction

Genetic mutations in the tryptophan‐aspartate repeat domain 72 (*WDR72)* gene result in the formation of enamel that is normal in thickness, but severely hypomineralized^1–3^. Previous studies have shown WDR72 loss of function in the enamel-producing ameloblast cells to result in enamel matrix protein retention and enamel hypomineralization^1,3^. These findings suggest that WDR72 regulates the removal of extracellular enamel matrix proteins in order for complete mineralization to occur.

Enamel formation is largely comprised of two major stages: secretory and maturation. During the secretory stage, mineralization is initiated by ameloblasts that secrete matrix proteins to establish an extracellular scaffold for newly formed hydroxyapatite crystals. In the following maturation stage, this protein scaffold is hydrolyzed and the extracellular proteins are endocytosed, to allow for crystal growth and final mineralization of the enamel matrix^4^. KLK4 (kallikrein related peptidase 4) is a well-established protease that is primarily responsible for protein hydrolysis of these extracellular matrix proteins during the maturation stage of enamel formation^5^.

Maturation stage ameloblasts are particularly unique in that they modulate between two morphologically and functionally distinct cell types, referred to as ruffle-ended (RE) ameloblasts and smooth ended (SE) ameloblasts. Unlike SE, RE have apical invaginations that are thought to help transport calcium into the matrix to form hydroxyapatite, which releases hydrogen ions to acidify the matrix (pH 5.8–6.2)^6^, possibly with additional acidification by plasma membrane associated H^+^ V-ATPase^7^. Ameloblasts then neutralize the matrix to pH 7.2 and lose their apical invaginations and modulate to SE. This acidification cycle continues until the matrix is fully mineralized and the tooth erupts.

A role of WDR72 in extracellular acidification has been shown by loss of maturation stage enamel matrix acidification in mice^2^, and renal tubular acidosis in humans^8,9^ in the presence of WDR72 mutations. GWAS studies have shown WDR72 to also be associated with executive functioning inhibition^10^ and diabetic retinopathy^11^. Finally, WDR72 has become a biomarker for some cancers^12–15^. These findings suggest a basic function for WDR72 in cellular process and regulation.

In this study, we present evidence that the functions of WDR72 in ameloblasts are related to regulation of microtubule structure and assembly. Microtubule assembly is critical for ameloblast modulation, endocytosis and vesicle trafficking required for pH regulation of the extracellular matrix. These results support a key role for vesicle trafficking and assembly in ameloblast-mediated enamel matrix mineralization.

## Results

### RNAseq pathway analyses showed WDR72 loss of function to be associated with extracellar matrix interactions, focal adhesion and P13-Akt signaling pathways

Differentially expressed genes (DEG) were identified in *Wdr72*^−/−^ as compared to *Wdr72*^+/+^ maturation stage enamel organs isolated from postnatal day 14 (P14) molars (NCBI Gene Expression Omnibus (GEO)). DEGs were subjected to analysis for GO biological process, Reactome, and KEGG^16^ pathways. The most significantly enriched DEGs in GO and Reactome pathways were extracellular matrix (ECM) structure and organization, while KEGG analysis showed significant enrichment in pathways related to protein digestion and absorption, ECM-receptor interaction, focal adhesion, and P13K-Akt signaling.

ECM and structural proteins found in the GO and Reactome enrichment pathways did not include enamel matrix proteins (such as amelogenin or enamelin), but instead included proteins associated with cell integrity. These proteins included collagen 8a2, which is associated with epithelial mesenchymal transformations^17^; collagen 23a1, a transmembrane protein, and collagens 4 and 6, which are networking collagens associated with the alterations in the basement membrane^18^. Among these analyses, differences related to structural integrity and focal adhesion pathways were an overarching theme, leading us to further investigate ameloblast structural changes.

### Ameloblasts from *Wdr72*^*−/−*^ mice have an incompletely formed ruffle border

**Figure 1:**
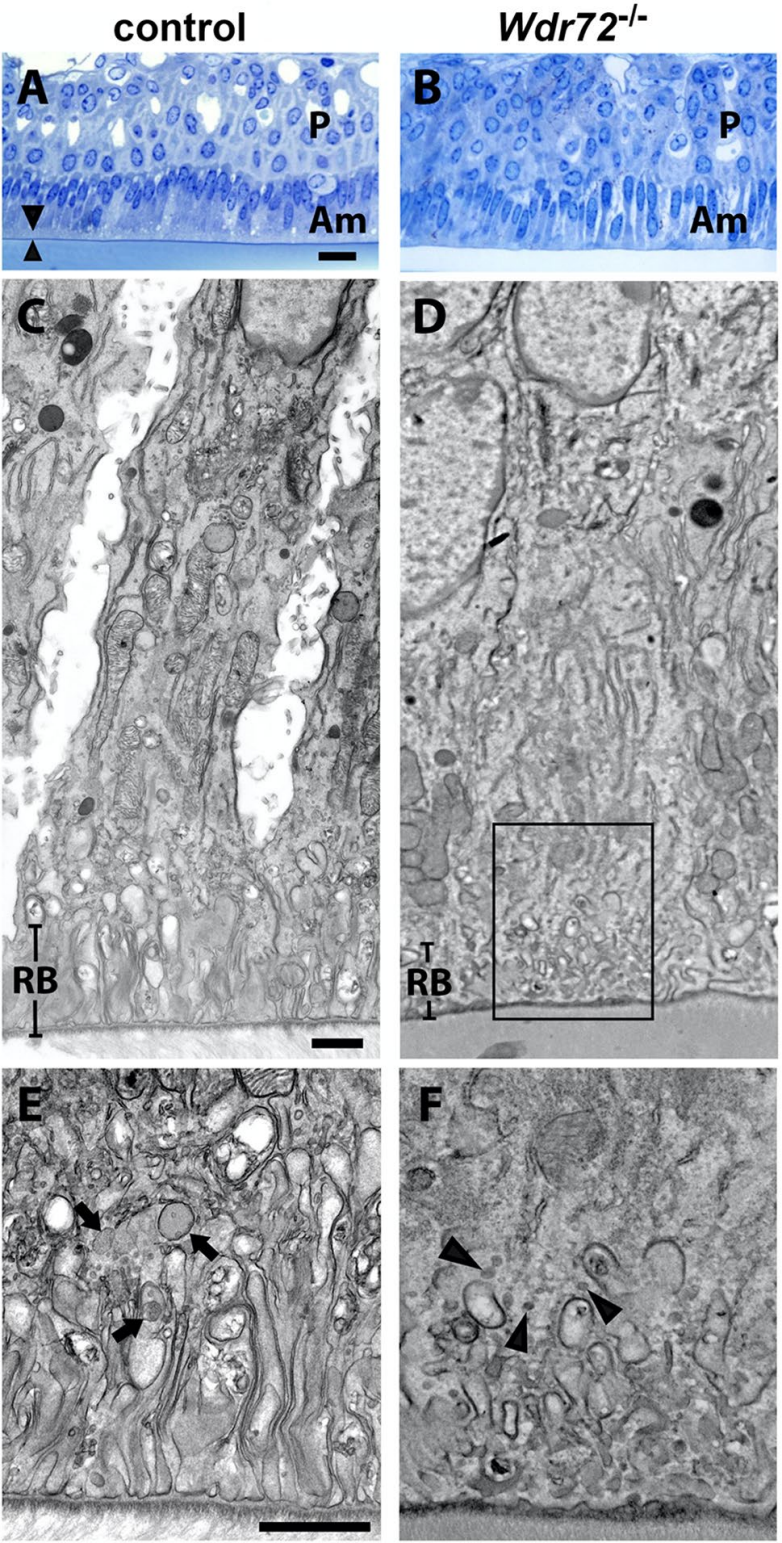
Ultrastructure of maturation-stage ameloblasts. (**A**,**B**) Light microscopic observation of the semi-thin sections of maturation ameloblasts immediately after the transition stage confirms the presence of the ruffled border (arrowheads) in control ameloblasts, while in *Wdr72*^*−/−*^ ameloblasts, the alignment of the ameloblasts layers is more disorganized, and the ruffled border is not evident. P: papillary layer, Am: ameloblasts, Bar: 20 µm. (**C**,**D**) Transmission electron micrographs show highly developed ruffled border (RB) in the control ameloblasts (**C**). In the *Wdr72*^*−/−*^ ameloblasts, only some irregular membrane invagination is observed at the distal end of the cells, and the distribution of the cytoplasmic organelles is disorganized (**D**), Bar 1 µm. (**E**,**F**) Insets of (**C**,**D**), respectively, in the area outlined by the black box. In the distal part of the control ameloblasts, small endocytic vesicles are fused and form larger early endosomes and multivesicular bodies (**E**, arrows), whereas in the *Wdr72*^*−/−*^ ameloblasts, small endocytic vesicles do not appear to fuse (**F**, arrowheads). Bar: 1 µm.

Comparison of maturation stage ameloblasts in *Wdr72*^+*/*+^and *Wdr72*^−/−^ ameloblasts by transmission electron microscopy (TEM) demonstrated differences in cell membrane integrity. *Wdr72*^+*/*+^ maturation stage ameloblasts showed a well-defined, polarized, and organized membrane forming distinct invaginations at the apical border of ruffle-ended ameloblasts (Fig. 1A,C,E). In contrast, *Wdr72*^−/−^ maturation ameloblasts, revealed a blunted and disorganized border (Fig. 1B,D,F). Additionally, vesicles were smaller in *Wdr72*^*−/−*^ ameloblasts (Fig. 1E,F; see black arrows), suggesting an inability to fuse and/or form vesicles in the cell cytoplasm^19^.

### Ex vivo proteinase activity and KLK4 mRNA expression was unaffected in *Wdr72*^*−/−*^ mice

**Figure 2:**
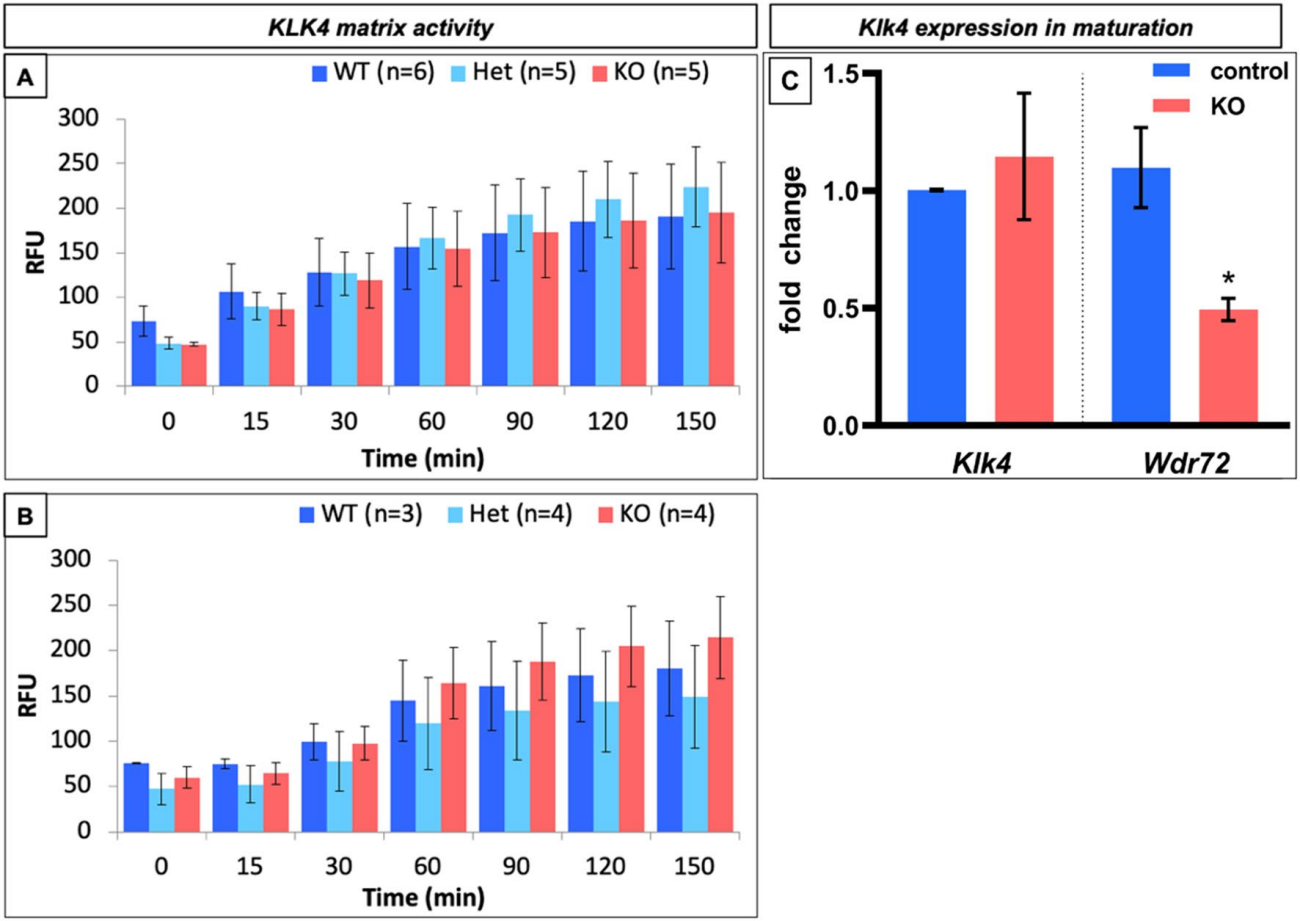
Proteinase activity, measured as relative fluorescence units (RFU) in the matrix extracts, normalized by weight of the tooth, showed no significant differences in matrix proteinase activity between *Wdr72*^+*/*+^*(WT)*, *Wdr72*^+*/−*^*(Het)*, and *Wdr72*^*−/−*^* (KO)* ameloblasts. (**A**) Incisors containing all stages of enamel matrix formation, and (**B**) maturation stage enamel matrix from postnatal day 14 (P14) molar had similar activity at all time points. (**C**) The right panel shows the relative quantitative real-time PCR (qPCR) of micro-dissected maturation-stage ameloblasts from first molars. Relative expression levels of *Klk4* and *Wdr72* were analyzed by the ddCt method. The expression of each gene was calculated as a fold change compared to the control. The significance of differences was determined by a paired t-test. P < 0.05 was considered significantly different.

Published data by Wang et al., shows higher molecular weight amelogenins in maturation stage enamel matrix isolated from *Wdr72*^*−/−*^ mice, as compared to controls^2^. This suggests an effect of WDR72 on matrix protein hydrolysis by the maturation stage proteinase, KLK4. However, we found no differences in either KLK4 activity in enamel matrix extracts from *Wdr72*^+*/*+^ and *Wdr72*^*−/−*^ mice (Fig. 2A,B), or in *Klk4* expression (Fig. 2C). It is possible, therefore, that the combined data suggest that WDR72 function is related to endocytosis or an alternate effect on KLK4 functionality is occurring.

### Endocytosis is delayed in *Wdr72*^*−/−*^ maturation-stage ameloblasts

**Figure 3:**
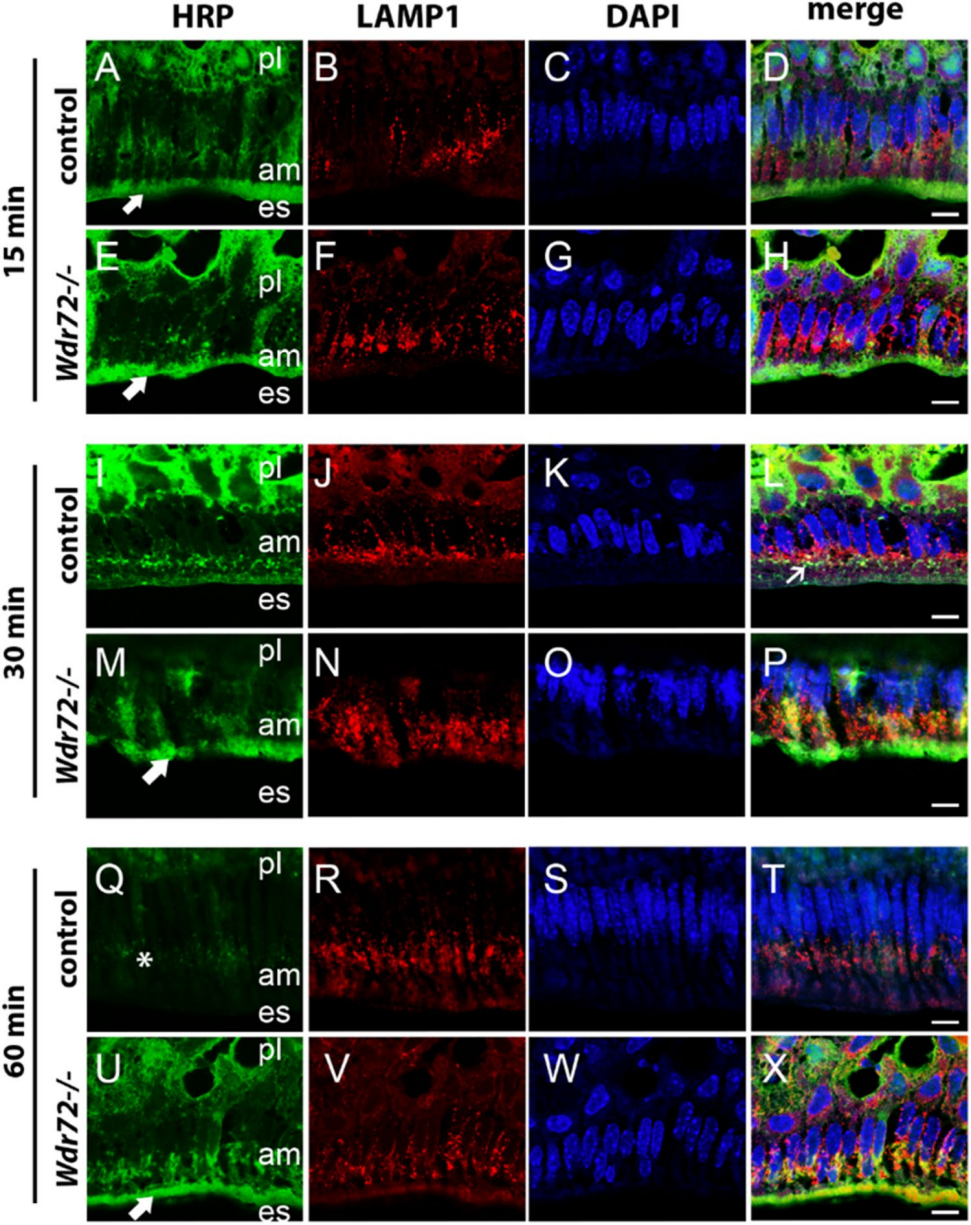
Localization of HRP and LAMP1 in control (*Wdr72*^+*/*+^ *and Wdr72*^+*/−*^) and *Wdr72*^*−/−*^ ameloblasts. Fifteen minutes following HRP injection, HRP localized to the apical border of both controls (**A**,**D**) and *Wdr72*^*−/−*^ ameloblasts (**E**,**H**). LAMP1 immunostaining (red), was apical to DAPI nuclear staining (blue). After 30 min, *Wdr7*^+*/*+^ mice showed HRP co-localized with LAMP1 positive vesicles (**I**,**L**), whereas Wdr72^−/−^ ameloblasts retained HRP at the apical border (**M**,**P**). Sixty minutes following injection, *control* cells showed lack of HRP presence, whereas *Wdr72*^*−/−*^ ameloblasts show delayed movement towards LAMP1 positive vesicles as well as continues HRP protein retention at the apical border.

Protein structure modeling suggested the possibility that WDR72 may be associated with membrane bending^3^, which is required for early endocytosis at the plasma membrane as well as formation and fusion of vesicles throughout the endocytic pathway. To determine whether endocytosis was altered by WDR72, we injected horseradish peroxidase (HRP) into control (*Wdr72*^+/+^ and *Wdr72*^+/−^) and *Wdr72*^−/−^ mice. We then localized HRP in relationship to LAMP1 vesicles, as a marker of lysosomes, at 15, 30 and 60 min time points. Similar to previous studies in wildtype rats^20^, controls showed classic HRP uptake patterns. In controls, after 15 min, HRP was taken up at the apical border of ameloblasts (Fig. 3A–D). At 30 min post-injection, controls showed some HRP co-localized with LAMP1, with much of the apical HRP absent (Fig. 3I–L). By 60 min, HRP was mostly absent in control ameloblasts (Fig. 3Q–T), presumably as it had been entirely digested by lysosomes. *Wdr72*^*−/−*^ ameloblasts showed similar initial HRP as controls (Fig. 3E–H). However, after 30-min, HRP remained largely localized to the apical border of *Wdr72*^*−/−*^ ameloblasts (Fig. 3M–P). After 60 min, *Wdr72*^*−/−*^ ameloblasts continued to show relatively high levels of HRP at the apical border with only minimal HRP co-localization with lysosomes (Fig. 3U–X). These results show a role for WDR72 in protein processing once inside the ameloblast cell, suggesting a role in vesicle trafficking.

### Ameloblast lineage cells (ALC) with CRISPR-Cas9 deletion of *Wdr72* exon 12 have reduced amelogenin uptake

**Figure 4:**
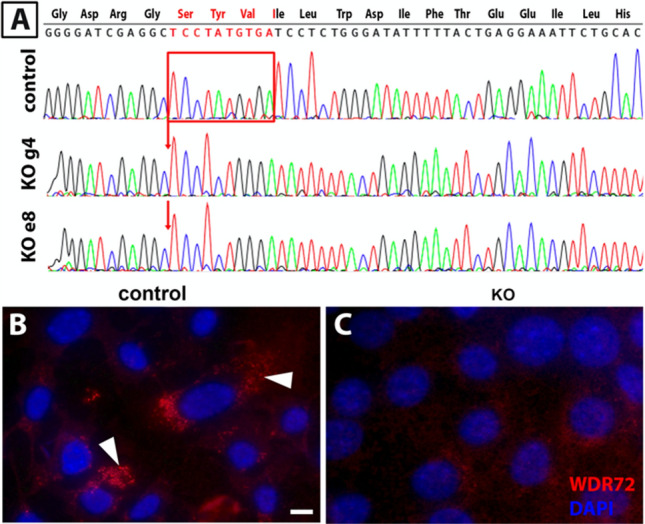
(**A**) Chromatograms of control and WDR72 deletion (KO*)* clones (**A**) show a 10-bp deletion (red box) that leads to a frame shift in the reading frame. (**B**) Immunostaining with anti-WDR72 (red) and counterstained with DAPI (blue) shows the presence of WDR72 in the cytoplasm of control cell (white arrowheads). (**C**) WDR72 is dramatically reduced in KO clones (**C**). Scale bar, 5 µm.

To further investigate uptake of amelogenin proteins by WDR72 loss of function ameloblasts, we used CRISPR-Cas9 to make WDR72 loss-of-function ALCs (ameloblast lineage cells)^21,22^. We targeted a 10-bp region within exon 12 of WDR72, causing a frameshift similar to deletions found in humans^3^ (see Fig. 4).

**Figure 5:**
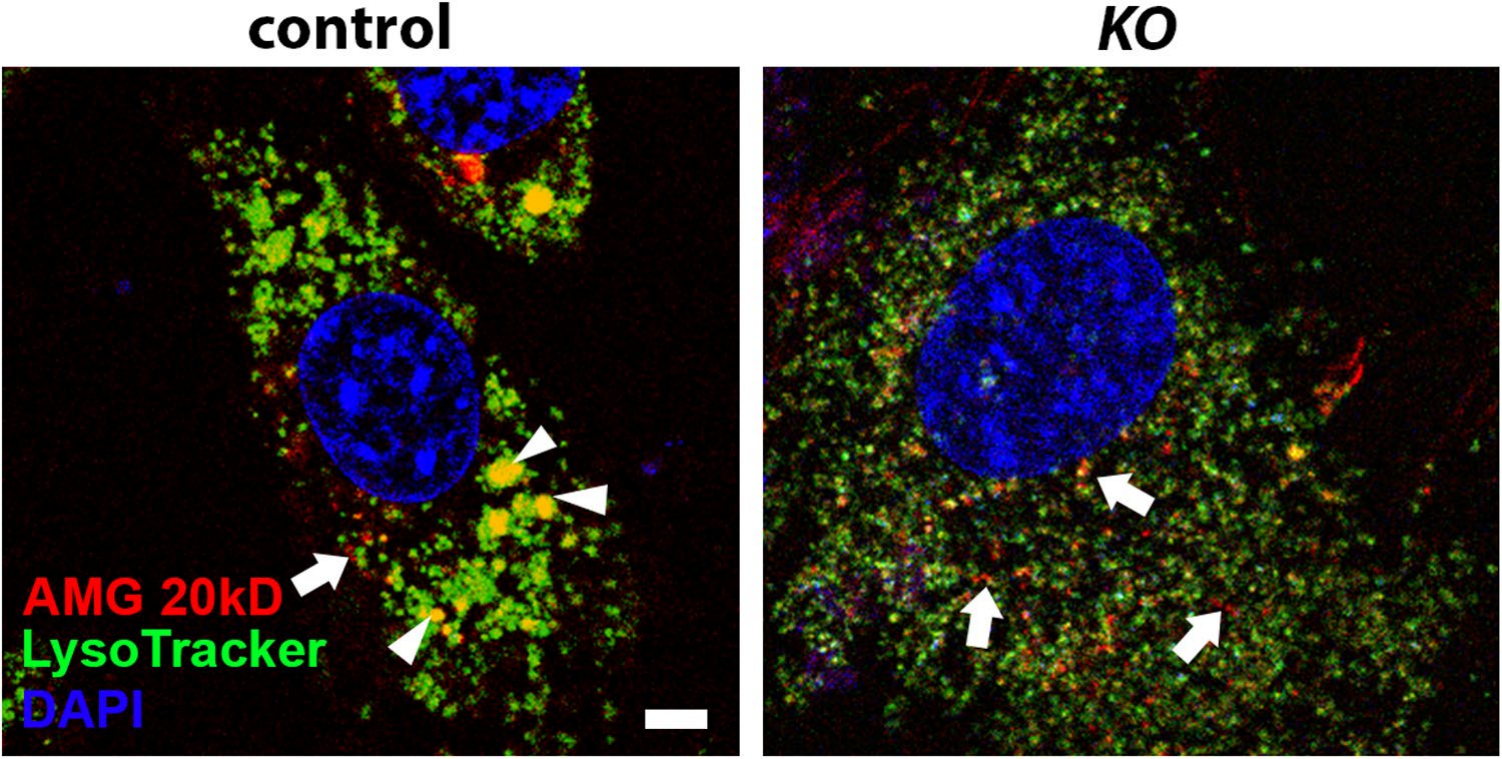
In control cells (left panel), rAMG20 (red) is taken up into the cells and co-localized (yellow) with acidic vesicles stained with LysoTracker (green) (white arrowheads). KO cells (right panel) showed numerous small, acidified vesicles stained green with Lysotracker dye. These vesicles did not form into larger sizes as seen in control cells, and while rAMG20 (red) is internalized into cells (arrows), apparent protein strands remain outside of the LysoTracker-positive vesicles. Scale bar, 2 µm.

To determine the function of WDR72 function in endocytosis of extracellular amelogenins, the predominant enamel matrix protein endocytosed by ameloblasts, we incubated control and KO ALC cell lines with red fluorescent labeled (Alexa Fluor 594) recombinant amelogenin 20 (rAMG20), followed by LysoTracker to identify acidified vesicles (lysosomes). We found that amelogenin was internalized by both control and KO cells. Control cells showed enlarged LysoTracker-positive vesicles that co-localized with rAMG20, indicating successful movement of the exogenous rAMG20 through the endocytoic pathway. However, in the KO cells, vesicles remained small and dispersed, akin to our TEM results (Fig. 2) and vesicular uptake of amelogenin was reduced (see Fig. 5).

### WDR72 does not contribute to vesicle acidification

**Figure 6:**
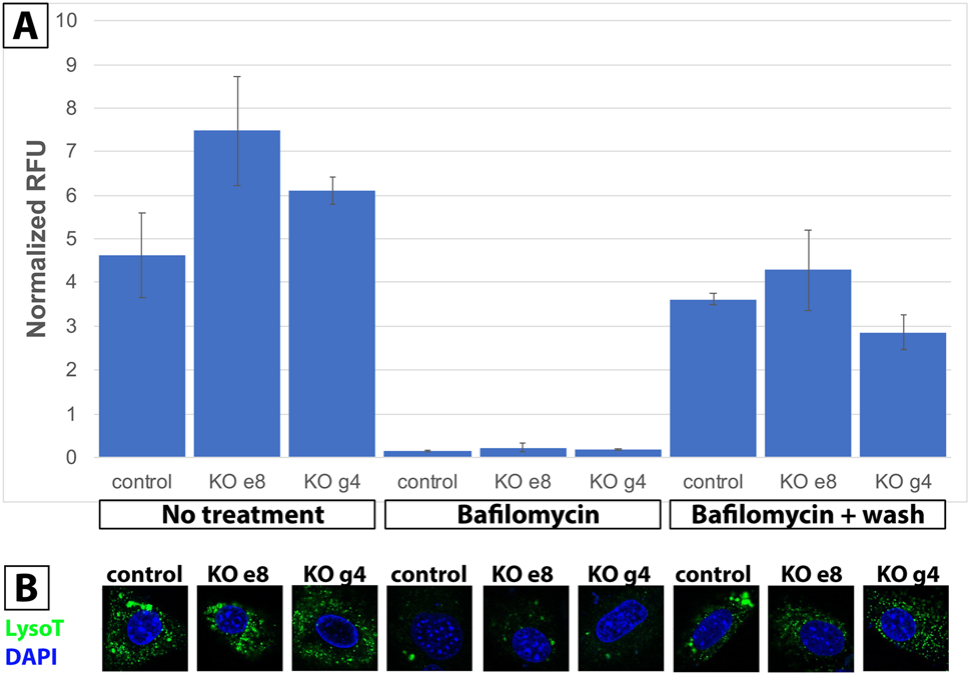
H^+^ v-ATPase dependent vesicle acidification. (**A**) Relative fluorescence units (RFU) measuring total lysotracker values normalized by DAPI show high values among control and KO clones (e8 & g4) at baseline. When V-ATPase inhibitor, bafilomycin, is added to the culture media, LysoTracker values significantly decrease across samples. After removing bafilomycin, all cells are able to return to re-acidifying their vesicles. (**B**) Confocal images of control and KO cells show LysoTracker staining (green) when no treatment and bafilomycin + wash is administered. Bafilomycin-treated cells show absent LysoTracker staining. Following the wash step, all cell types showed increased acidification as indicated by LysoTracker staining. In the no treatment and bafilomycin + wash groups, note the large aggregates of LysoTracker positive control cells relative to KO clones, which suggests the presence of larger-sized lysosomes in control cells only.

WDR7, the closest homolog to WDR72, is known to mediate endocytosis by regulating vesicle acidification by H^+^ V-ATPase^23^. To determine whether WDR72 related effects on endocytosis are by modulating vesicle acidification, we tested vesicle reacidification following treatment with the V-ATPase inhibitor, bafilomycin A1, as previously described for WDR7^23^. Both quantitative (Fig. 6A), and qualitative (Fig. 6B) analyses showed no effect of WDR72 on vesicle acidification. This finding shows that WDR72 regulates endocytosis independently of vesicle acidification.

### Tubulin was dysregulated in WDR72 loss of function cells

**Figure 7:**
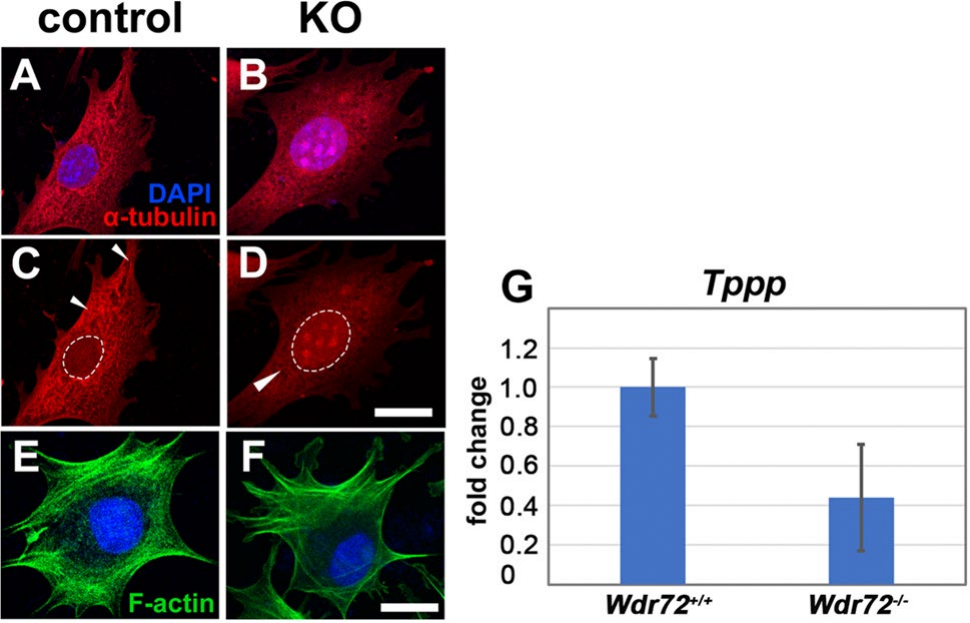
WDR72 function is linked to microtubule related structural compoments. Confocal microscopy sections of (**A**,**C**) control cells show alpha-tubulin (red) localized intracellulary in an organized, web-like pattern in the cytoplasm with organized focal points at the border of cells (**C**; arrowheads). (**B**,**D**) KO cells exhibited diffuse alpha tubulin intracellular staining and localization to the nucleus, without organized aggregates at the cell border. (**A**,**B**) show the nucleus overlain by blue DAPI staining, and (**C**,**D**) show the location of the nucleus with a white dotted outline. In contrast, F-actin (green) shows typical web-like striations throughout the cell that organize around the nucleus (blue) in both (**E**) control and (**F**) KO cells. (**G**) The average expression level of *Tppp* in *Wdr72*^*−/−*^ P13 molar enamel organ was 44% that of *Wdr72*^+*/*+^ P13 molar enamel organ (*P = 0.04).

To investigate the role of WDR72 in focal adhesion pathways as indicated in our GO/KEGG analyses, we immunostained control and KO ALCS for alpha-tubulin and F-actin. Control cells showed typical immunostaining for alpha-tubulin, with a web-like pattern in the cytoplasm and focal adhesion aggregates along the cell borders. However, KO ALCs showed alpha-tubulin immunolocalization to the nucleus, and more diffuse cytoplasmic staining (Fig. 7A–D), while F-actin, however, appeared unaffected (Fig. 7E,F). RNAseq showed significantly decreased tubulin polymerization promoting protein (TPPP) in *Wdr72*^*−/−*^ ameloblasts, which we confirmed by qPCR analyses of maturation-stage ameloblasts (Fig. 7G).

**Figure 8:**
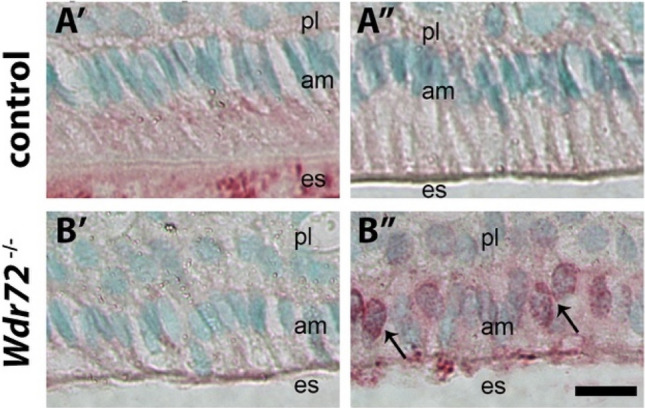
ANXA8 (red) is present in early maturation stage ameloblasts in (**A′**) control (*Wdr72*^+*/*+^ *and Wdr72*^+*/−*^) ameloblasts, while largely absent in *Wdr72*^*−/−*^ ameloblasts (**B′**). Later in the maturation stage, ANXA8 is not apparent in control ameloblasts (**A″**). However, in *Wdr72*^*−/−*^ ameloblasts, ANXA8 is localized around the nucleus (**B″**, arrows). am, ameloblast; es, enamel space; pl, papillary layer. Scale bar, 10 μm.

To further investigate the possibility of WDR72 related effects on microtubules in vivo, we immunostained for annexin A8 (ANXA8). ANXA8 interacts with microtubules in association with the maturation of late endosomes to multivesicular bodies (MVBs) and lysosomes^24,25^. We found reduced ANXA8 immunostaining in *Wdr72*^−/−^ in early maturation stage ameloblasts, as compared to *Wdr72*^+*/*+^ controls. Later in maturation, ANXA8 was immunolocalized in the perinuclear region of *Wdr72*^−/−^ ameloblasts, but was largely absent in controls (Fig. 8).

## Discussion

Tooth enamel formation is a highly regulated process involving a dynamic relationship between the ameloblast cells and the extracellular matrix. A key to understanding this highly organized mineralization process is the function of WDR72, a gene known to be critical for enamel maturation. Enamel matrix proteins are retained in *Wdr72*^*−/−*^ enamel matrix, suggesting a delay in matrix protein hydrolysis, which is required for mineral formation. In support of this possibility, data published by Wang et al.^2^ suggested reduced amelogenin hydrolysis in the *Wdr72*^*−/−*^ enamel matrix.

We found that KLK4, a serine proteinase which is primarily responsible for hydrolyzying maturation stage enamel matrix proteins, had similar activity in matrix protein extracts from *Wdr72*^+*/*+^ , *Wdr72*^+*/−*^*,* and *Wdr72*^*−/−*^ mice. The similar KLK4 mRNA expression and KLK4 activity in enamel matrix extracts in all *Wdr72* mouse models, indicates that WDR72 does not affect KLK4 exocytosis into the enamel matrix. However, instead, because KLK4 is maximally active at pH 6^26^, it is possible that KLK4 activity is reduced in situ in the neutral (pH 7.2) *Wdr72*^*−/−*^ matrix, which unlike control enamel does not cycle to an acidic pH (pH 6.2)^2^.

Enamel matrix protein retention indicates a role of WDR72 in endocytosis^1,3^. Our studies which tracked HRP uptake into ameloblasts in vivo, and recombinant amelogenins into acidified vesicles in vitro, confirm a role of WDR72 in matrix protein endocytosis. Previous studies of WDR72 speculated that WDR72 may regulate endocytosis in a manner similar its closest human homolog, WDR7^3,27^. WDR7 regulates endocytosis through its role in acidification of H^+^ v-ATPase^23^. We found vesical acidification was not altered with WDR72 loss of function, and therefore WDR72 has a unique function, different from that of WDR7, in modulating cellular endocytosis.

TEM imaging of the *Wdr72*^*−/−*^ ameloblasts showed a dysregulated apical border, and changes in vesicle formation. These multiple phenotypes of WDR72 loss of function ameloblasts, combined with our RNAseq analyses showing an effect of WDR72 on focal adhesion pathways and P13k-Akt signaling, point to an effect of WDR72 on microtubule formation^28^. F-actin function in focal adhesion attachment to integrins on the cell surface, is mediated by microtubules^29,30^, and P13k-Akt signaling can promote microtubule stabilization^28^.

To further determine the effects of WDR72 on F-actin organization and microtubule assembly, we used CRISPR-Cas9 to generate WDR72 loss of function ameloblast lineage cells (ALCs). Immunostaining of KO and control cells showed increased alpha tubulin immunolocalization to the cell nucleus in WDR72 KO cells, and no obvious changes in F-actin localization. These findings point to a dysregulation of microtubule assembly in *Wdr72* KO ameloblasts. Such microtubule dysreguations are consistent with our findings of poorly formed ameloblast apical borders, delayed endocytosis, and vesicle trafficking required for calcium transport^2^ and regulation of matrix pH^2^.

Microtubules organize into centers in the nucleus during mitosis, and are also well-known to generate within the cytoplasm^31^. Tubulin is exported from the nucleus through the action of exportin1/CRM1^32^. Our RNAseq data showed no changes in relative expression of exportin1/CRM1 mRNA in *Wdr72*^*−/−*^ ameloblasts as compared to controls*.* However, dynein, a protein that interacts with microtubules in the nucleus to modulate nuclear rotation^33^, was significantly upregulated in *Wdr72*^*−/−*^ ameloblasts. Loss of WDR72 may increase microtubule stabilization, as indicated by the significant increase in microtubule associated protein 1 (MAP1), a protein known to stabilize microtubules^34^, and P13k-Akt signaling, which also promotes microtubule stabilization^28^. Consistent with these finding, RNAseq and qPCR amplification, showed significantly reduced expression of TPP1 (tubulin polymerization promoting protein 1) in *Wdr72*^*−/−*^ ameloblasts. TPP1 is a regulator in microtubule dynamics by driving microtubule polymerization^35^.

ANXA8, which interacts with microtubules to regulate late endosome organization and function^25^ was also dysregulated in *Wdr72*^*−/−*^ ameloblasts. Movement of late endosome/multivesicular body (LE/MVB) to lysosomes (LYS)^36,37^ is mediated by microtubules, which guide membrane turnover^38^. Therefore, the lack of ANXA8 immunostaining in early maturation stage *Wdr72*^*−/−*^ ameloblasts, with increased staining later in maturation, as compared to control ameloblasts, is consistent with a delay in the formation of late endosomes through altered microtubule interactions.

Regardless of the exact mechanism by which WDR72 may direct microtubule assembly or stabilization, these results have important implications for understanding the effects of WDR72 in cells. For example, microtubule structural changes are critical for neuronal functioning^39–41^, which could explain the role of WDR72 in executive functioning^10^. And, microtubules are essential for the recycling of ion transporters necessary for renal acidification, which could explain the role of WDR72 in renal acidosis. The study of WDR72 function in ameloblasts highlights the usefulness of this model system, and how endocytosis, vesicle assembly, ion transport and modulation of the plasma membrane, are integrated to direct enamel matrix mineralization.

## Experimental procedures

This study is reported in accordance with ARRIVE guidelines^42^. All procedures involving animals were reviewed and approved by the IACUC committee at the University of California, San Francisco, in compliance with the guidelines in the revised Animals (Scientific Procedures) Act 1986. Heterozygous mice (*Wdr72*^+/−^) on a C57BL/6 genetic background were bred to generate the knockout (*Wdr72*^−/−^) and control (*Wdr72*^+/+^) mice used in these studies. As previously described^3^, genotypes of mice were determined by standard and quantitative PCR (Transnetyx, Cordova, TN) using genomic DNA obtained from tail biopsies with forward primers: *NeoF*-GGGATCTCATGCTGGAGTTCTTCG, *F*-TCTTTCACCTAAGCAACACATGCGG, and reverse primer *R*-GAAACCCGGAGATGAAGGAATGTGC. Amplicon sizes of *Wdr72*^+^ and *Wdr72*^−^ alleles were 520 and 633 bp, respectively.

### RNAseq

After genotyping, mouse pups at postnatal 12 days old (P12) were euthanized with CO_2 _inhalation by following the standard IACUC protocols. The first molars were removed and rinsed in cold PBS. The molars were treated by Trizon reagent (Invitrogen) for 5 min to lyse the enamel organ cells overlaying on the first molars. Total RNA of enamel organ cells was collected by using Direct-Zol RNA miniprep Kit (Zymo Research). RNA from five pups was pooled as one sample. Four biological replicates from each *Wdr72*^+*/*+^ or *Wdr72*^*−/−*^ mouse model were subjected to RNA (whole transcriptome) sequencing. As a result, 50 million paired-end reads per sample were obtained. The average of Person Correlation Coefficient between biological replicates, an important parameter to validate reliability of sample collection and repeatability of the experiment, was about 97%. The base error rate of all sequences was 2%.

To identify genes regulated by the presence of WDR72, DESeq2 R package was utilized to run the statistical analysis, and the resulting P values were adjusted using the Benjamini and Hochberg's approach for controlling the false discovery rate. These DEGs were subjected to GO biological process, Reactome, and KEGG pathway enrichment analyses.

### Histomorphometry of maturation stage ameloblasts

For TEM imaging, 2 month old male and female littermates (n = 2) were anesthetized in tribromoethanol in 0.9% NaCl and perfused with fresh 3% paraformaldehyde and 2.5% glutaraldehyde in 0.06 M cacodylate buffer (pH 7.3). Mandibles and maxillae were dissected and further immerse-fixed in the same fixatives at 4 °C overnight. Following rinsing in 0.06 M cacodylate buffer, the mandibles and maxillae were decalcified in 8% EDTA (pH 7.3) supplemented with 1% glutaraldehyde at 4 °C under constant agitation for 2 weeks. Samples were then post-fixed with 1% osmium tetroxide in 0.06 M cacodylate buffer (pH 7.3) for 2 h at 4 °C and dehydrated through graded ethanol and embedded in LR white acrylic resin (London Resin Company, Reading, UK). One-micrometer-thick semi-thin sections were obtained for Toluidine Blue staining and light microscopic observation. An area where ameloblasts were immediately after the transition stage was chosen for ultrathin sectioning (80–100 nm). The ultrathin sections were placed on FormvarTM- and carbon-coated nickel grids, double stained with UAR-EMS Uranyl Acetate Replacement Stain (Electron Microscopy Sciences, Hatfield, PA) and 3% lead citrate, and examined under the FEI Tecnai G2 transmission electron microscope (Thermo Fisher Scientific Electron Microscopy, Hillsboro, OR) at an acceleration voltage of 80 kV.

### KLK4 activity assay

Mandibular and maxillary first molars of *Wdr72*^+*/*+^, *Wdr72*^+*/−*^*, and Wdr72*^*−/−*^ post-natal day 14 littermates were dissected free of alveolar bone, the enamel organs removed under a dissecting microscope, and 4 molars from each mouse were combined together, weighed, and placed in 0.5 M acetic acid overnight at 4 °C. Incisors dissected from 1 year old littermates were separately weighed, and similarly placed in 0.5 M acetic acid overnight at 4 °C. Total protein in the acetic acid extracts was measure by BCA assay, and relative KLK4 proteolytic activity was quantitated by measuring digestion of a fluorogenic peptide substrate with a KLK4 specific cleavage site (Boc-V-P-R-AMC, R&D Systems) using a SpectraMax iD3 fluorescence plate reader (Molecular Devices) at excitation of 380 nm and emission at 460 nm. Fluorescent readouts measuring KLK4 activity were recorded every minute for 150 min. All measurements were standardized using corresponding sample weights.

### Endocytosis of horseradish peroxidase (HRP)

One month old male and female *Wdr72*^+*/*+^, *Wdr72*^+*/−*^*,Wdr72*^*−/−*^ littermates (n = 5) were anesthetized subcutaneously with tribromoethanol, and 7 units (0.7 mL) of 5% HRP *(Sigma-Aldrich, MO)* (P9250-25KU) in 0.9% saline were injected into the jugular vein through the deltopectoral groove at the intersection of the jugular and axillary veins via a 31 gauge needle at a rate of 7 units per minute. 0.9% saline solution was injected into control mice serving as a negative control. Mice were kept under general anesthesia for 15, 30 or 60 min, prior to euthanasia and collection of maxillary and mandibular incisors. Following HRP injections, both mandibular and maxillary jaws were removed of all soft tissue and incisor tips and cervical loops removed to fit into cryomolds. Samples were then incubated twice in 10 mL of 30% sucrose at room temperature until sinking (approximately 15 min). They were next placed in OCT medium to acclimate and embedded into OCT-containing cryomolds in a similar orientation for cryosectioning. Cassettes were placed in a dry ice and pure hexane mixture to freeze tissue and stored in − 80 °C until ready for cryosectioning. Cryosections were performed at − 17 °C chamber temperature and sectioned at 7 µm to be placed on CryoJane slides. Once affixed to slides, sections were rehydrated in PBS for 5 min and immunostained for lysosomal-associated membrane protein 1 (LAMP1), a common marker of lysosomes, as well as with TSA fluorescein at 1:50 dilution per manufactures instructions. Sections were then counterstained with Hoescht DAPI stain (Invitrogen, H3570) at 1:10,000 dilution for 15 min and imaged under confocal microscopy.

### CRISPR/Cas9 WDR72 deleted ameloblast lineage cells (ALCs)

ALCs of passage 22 were generously provided as a gift from Dr. Toshihiro Sugiyama, Akita University, Japan, and Dr. John Bartlett, The Ohio State University. The cells were grown to 70% confluence, then then incubated at 37 °C for 72 h in serum-free media, 2.5 µM sgRNA transfection complex, 100 ng/µL Edit-R EGFP fluorescent Cas9Nuclease mRNA (Dharmacon, CAS11860), and transfection reagent. sgRNA transfection complexes (crRNA: tracrRNA in 10 mM Tris Buffer, pH 7.4) were formed using either Edit-R crRNA Non-targeting Control #1 (U-007501-01-05) as a negative control, Edit-R-Ppib synthetic crRNA Control (U-007100-01-05) as a positive control targeting GTATACTTTGATTTACAAAT sequence, or Edit-R Mouse *Wdr72* (546144) crRNA (CM-068518-01-002) targeting AATATCCCAGAGGATCACAT at mm10|-chr9: 74155036–74155058 AGG, Accession NM_001033500.3 (exon 12), combined with Edit-R CRISPR-Cas9 Synthetic tracrRNA (U-002005-20).

Following treatment with CRISPR/Cas9, cells were grown to 80% confluence in DMEM supplemented with 10% FBS, 1% penicillin, and 1% streptomycin. Following rinsing, trypsinization, and re-suspension, cells were single-cell sorted at the PFCC flow cytometry core facility at Parnassus, UCSF (DRC Center Grant NIH P30 DK063720), using the Aria Fusion machine (NIH S10 1S10OD021822-01). Cells were gated for green fluorescence protein (GFP) and viability and subsequently plated as single-cells in 96-well plates. For the following 2 weeks, cells were monitored for single-colony growth in culture media and expanded for DNA sequencing verification. DNA was first purified using QIAquick PCR Purification Kit (Qiagen Sciences, Louisville, KY) (28104), and verification of cell knockout (KO) lines were performed using Sanger Sequencing.

### Quantitative fluorescence-based vesicle acidification assay and imaging

Control and KO cell clones were grown on either 96-well plates or 35 mm glass bottom dishes No. 0 (MatTek) (P35GC-0-14-C/H) to undergo one of the following groups: no treatment (media only), 50 nM bafilomycin, or 50 nM bafilomycin plus a 3-h washout. Cells in 96-well plates were used for quantification of fluorescence, while those in dishes were imaged under confocal microscopy for qualitative assessment.

Control and KO cells used for quantification were incubated with either media only, 50 nM bafilomycin A1 (Sigma-Aldrich, MO) (SML1661) for 1 h, or 50 nM bafilomycin A1 for 1 h that was then washed out for 3 h. All cell groups were labeled with a mixture of DAPI and LysoTracker for 10 min, then rinsed three times in PBS and lysed in 200 µL of RIPA lysis buffer (50 mM Tris–HCL, pH 7.4; 150 mM NaCl, 1% NP-40, 0.5% sodium deoxycholate, 0.1% SDS). The lysate was then transferred into a black 96-well plate (Corning, Corning, NY), and fluorescence released into the medium was immediately read using a SpectraMax iD3 fluorescence plate reader (Molecular Devices) with 0.4-s integration time, at 500/565 nm for LysoTracker and 350/461 nm for DAPI. Total green fluorescence values (number of vesicles) were normalized to total blue values (cell number), and then to the untreated control (media only). Statistical significance was determined by one-way analysis of variance (ANOVA). *P-*values < 0.05 were considered significant.

Control and KO cells used for confocal imaging were performed in quadruplicate biological replicates incubated under the same conditions as quantitative studies performed and imaged using an inverted confocal microscope (Leica Microsystems, DMi8) with Leica Application Suite X imaging software and processed using Fiji plug-ins and ImageJ^43^.

### Endocytosis of recombinant amelogenin in vitro

20kDA recombinant amelogenin (rAMG 20kD) was synthesized and purified as previously described^44^. The purified rAMG 20 kD was labeled with Alexa Fluor 594, using a Microscale Protein Labeling Kit (Thermo Fisher Scientific, MA) (A30008).

Control and KO cell clones were grown to 70% confluence at equivalent cell densities on 35 mm glass bottom dishes No. 0 (MatTek) (P35GC-0-14-C/H), then starved in serum-free media for 24 h to facilitate endocytosis. Following starvation, 1 µL of 1 mg/mL labeled rAMG 20kD was added to media for 30 min. A solution of Hoescht DAPI stain (Invitrogen, H3570) and LysoTracker Green DND-26 (Life Technologies, CA, L7526) in 1× PBS/FBS were incubated with cells for 5 min. All cell groups were rinsed 3 times, and imaged under confocal microscopy for 45, 60, 90, and 135 min after rAMG 20kD was added.

### Immunolocalization of alpha-tubulin and F-actin in vitro

Control and *Wdr72* KO cell clones were grown to 70% confluence and rinsed with microtubule stabilizing buffer (MSTB) with 0.5% glutaraldehyde for 30 min. Cells were then permeabilized with MSTB/0.5% Triton X at 25 °C for 60 min, rinsed 3 times with PBS, and labeled with primary mouse anti-chick tubulin antibody (Thermo Fisher Scientific, MS-581, DM1A). M.O.M. biotinylated anti-mouse IgG/M.O.M. diluent was used as the secondary antibody for 10 min, followed by streptavidin 594 (DYlight, SA5594) at 1:100 dilution in the dark for 30 min. Phalloidin AF 488 green was used to label F-actin and was incubated with cells for 20 min in the dark. Cells were then counterstained with Hoescht DAPI stain (Invitrogen, H3570) at 1:10,000 dilution for 15 min and imaged under confocal microscopy.

### Immunolocalization of annexin 8A in vivo

Mandibles from *Wdr72*^+*/*+^, *Wdr72*^+*/−*^*,* *Wdr72*^*−/−*^ litermates (n = 4) were immediately immerse-fixed in 4% paraformaldehyde (PFA)/0.06 M cacodylate buffer (pH 7.3) overnight and decalcified in 8% EDTA (pH 7.2) for 2 weeks at 4 °C. Samples were then dehydrated and paraffin-processed for routine embedding and sectioning. Sagittal incisor sections at 5 μm were deparaffinized and rehydrated, followed by incubation with 10% swine serum for blocking. Rabbit polyclonal antibody to annexin-A8 (Thermo Fisher Scientific) (PA5-31479) was used at 1:500 dilution. Following overnight incubation at 25 °C, sections were washed a biotinylated secondary antibody (Dako, Carpinteria, CA) was added for 1 h at 25 °C. Alkaline phosphatase conjugated to streptavidin (Vector Laboratories Inc., Burlingame, CA) was used to visualize the colorimetric reaction. Sections were then counterstained using methyl green (Dako, Carpinteria, CA).

### qPCR

Maxillary and mandibular whole first molars were dissected from post-natal day 10 *Wdr72*^+*/*+^ and *Wdr72*^*−/−*^ mice siblings. Ameloblasts at this stage have enamel primarily at the maturation stage, and they were microdissected from the surface of the teeth. Total RNA was purified, and qPCR was performed to determine the relative amounts of *Klk4* and *Tppp* (tubulin polymerization promoting protein) mRNA in *Wdr72*^+*/*+^ and *Wdr72*^*−/−*^ mice. Samples were run with 18S as the endogenous control. The primer sequences for the Klk4 gene were as follows: sense-GCTGGATGACAACCTGCTGTACT, antisense-TGTGGGACCGGCTTCATG. The primer sequences for the *Tppp* gene were as follows: sense—AGGGCTGCTAAGAGGTTGTCA, antisense—GGTGTCCCCATGTACTGCAA.
